# Spill-Over from Public Health? First Detection of an OXA-48-Producing *Escherichia coli* in a German Pig Farm

**DOI:** 10.3390/microorganisms8060855

**Published:** 2020-06-05

**Authors:** Alexandra Irrgang, Natalie Pauly, Bernd-Alois Tenhagen, Mirjam Grobbel, Annemarie Kaesbohrer, Jens A. Hammerl

**Affiliations:** 1 Department Biological Safety, Unit Epidemiology, Zoonoses and Antimicrobial Resistance, German Federal Institute for Risk Assessment (Bundesinstitut für Risikobewertung, BfR), D-10589 Berlin, Germany; natalie.pauly@bfr.bund.de (N.P.); bernd-alois.tenhagen@bfr.bund.de (B.-A.T.); Mirjam.Grobbel@bfr.bund.de (M.G.); Annemarie.kaesbohrer@bfr.bund.de (A.K.); jens-andre.hammerl@bfr.bund.de (J.A.H.); 2 Institute for Veterinary Public Health, University of Veterinary Medicine, 1210 Vienna, Austria

**Keywords:** OXA-48, CPE, carbapenemase, food chain

## Abstract

Resistance to carbapenems is a severe threat to human health. These last resort antimicrobials are indispensable for the treatment of severe human infections with multidrug-resistant Gram-negative bacteria. In accordance with their increasing medical impact, carbapenemase-producing Enterobacteriaceae (CPE) might be disseminated from colonized humans to non-human reservoirs (i.e., environment, animals, food). In Germany, the occurrence of CPE in livestock and food has been systematically monitored since 2016. In the 2019 monitoring, an OXA-48-producing *E. coli* (19-AB01443) was recovered from a fecal sample of a fattening pig. Phenotypic resistance was confirmed by broth microdilution and further characterized by PFGE, conjugation, and combined short-/long-read whole genome sequencing. This is the first detection of this resistance determinant in samples from German meat production. Molecular characterization and whole-genome sequencing revealed that the *bla*_OXA-48_ gene was located on a common pOXA-48 plasmid-prototype. This plasmid-type seems to be globally distributed among various bacterial species, but it was frequently associated with clinical *Klebsiella* spp. isolates. Currently, the route of introduction of this plasmid/isolate combination into the German pig production is unknown. We speculate that due to its strong correlation with human isolates a transmission from humans to livestock has occurred.

## 1. Introduction

Carbapenem resistance by the production of carbapenemases is increasingly reported from medical settings [1]. In humans, OXA-48 is the most frequent carbapenemase in Germany [1]. In 2018, its occurrence was mainly associated with *Klebsiella* species, but also with the *E. coli* (25% of detected isolates). In 2001, OXA-48 was detected in Turkey for the first time and subsequently reported from the Mediterranean and Western Europe in the following years [2,3,4,5]. Nowadays, this enzyme variant is globally disseminated [6]. The resistance is mediated by the *bla*_OXA-48_ gene, which was supposed to originate from the genus *Shewanella* [7]. The gene is typically located on a highly conserved plasmid-prototype (pOXA-48) carrying an IncL/M replicon [8].

It is well known that the use of carbapenems has supported the distribution of carbapenemase-producing Enterobacteriaceae (CPE), especially of invasive *Klebsiella pneumoniae*, in humans in recent years [9]. In Europe, carbapenems are not licensed for use in veterinary medicine. Nevertheless, CPE have been sporadically detected in livestock, companion animals, and wildlife in recent years [10,11]. In Germany, VIM-1-producing *E. coli* and *Salmonella* sp. were repeatedly recovered from the German pig production and from pork since 2012 [12,13]. Although OXA-48 is the most common enzyme variant in CPE in humans, no OXA-48 producing Enterobacteriaceae was reported before. In 2019, first *bla*_OXA-48_-carrying *E.coli* was detected from German livestock. This study aims to provide a comprehensive characterization of the isolate as well as deep whole genome information data in order to assess the impact of the isolate for public health and identify a potential novel target plasmid for the dissemination of carbapenem-resistance.

## 2. Materials and Methods

### 2.1. Isolate Origin

Within the German monitoring on CPE the *E. coli* strain 19-AB01443 was isolated from a fecal sample of fattening pigs at farm in Germany. The farm was a farrow to finish operation that did not routinely purchase animals but raised its own replacement gilts. The sample was subjected for cultivation by the regional state laboratory according to the EURL-AR protocol (https://www.eurl-ar.eu/protocols.aspx; 10.01.2020). The recovered isolate was sent to the National Reference Laboratory for Antimicrobial Resistance (NRL-AR) hosted at the German Federal Institute for Risk Assessment for confirmatory-testing and in-depth characterization. 

### 2.2. Phenotypic and Genotypic Isolate Characterization

Growth of the *E. coli* isolate (19-AB01443) on selective CPE agar was confirmed using chromID^®^ CARBA SMART agar plates (Biomerieux, Nürtingen, Germany). Minimal inhibitory-testing (MIC) of the isolate was conducted using broth microdilution procedure following the CLSI guidelines (version A7-M11). The MIC values were interpreted according to epidemiological cutoff values that were provided by EUCAST. The presence of the carbapenemase gene and phylogenetic group of the isolate were confirmed by real-time PCR and PCR analysis, respectively [14,15]. PFGE was carried out according to PulseNet protocol (https://www.cdc.gov/pulsenet/pathogens/protocols.html). The location of the *bla*_OXA-48_ was analyzed by S1-PFGE with subsequent Southern-blotting and DNA-DNA hybridization against a digoxigenin-labelled *bla*_OXA-48_ probe while using DIG EasyHyb and DIG Wash and Block Buffer Set (Roche Diagnostics, Mannheim, Germany) according to the manufacturer recommendations. The PBRT Kit 2.0 (Diatheva, Fano, Italy) was further used to determine the replicon-type of the *bla*_OXA-48_-carrying plasmid. The transmissibility of the plasmid was investigated by filter-mating assays with different wildtype Enterobacteriaceae as potential hosts (*E. coli, Citrobacter freundii, Enterobacter asburiae*, *Klebsiella oxytoca* and *Salmonella* Typhimurium) [16]. The pOXA-48 EC-1443 plasmid was further transformed into highly competent *E. coli* DH10B bacteria (ElectroMAX^TM^ DH10B^TM^ Cells; Invitrogen^TM^, Thermo Fisher Scientific, Schwerte, Germany) by electroporation using disposable cuvettes with 1 mm gap and, 1.8 kV (E = 18 kV/cm) in a Bio-Rad MicroPulser (Bio-Rad Laboratories, Feldkirchen, Germany). For in-depth characterization, the *E. coli* isolate was subjected to whole-genome sequencing on both a short-read- (NextSeq, Illumina, San Diego, CA, USA) and on a long-read-generating platform (MinIon, Oxford Nanopore, UK) as recommended by the manufacturers. The raw reads from both sequencing platforms were subjected to a hybrid assembly (unicycler, v.0.44). The hybrid assemblies of the *E. coli* isolate 19-AB01443 and the pOXA-48EC-1443 plasmid were deposited in GenBank (NCBI) under the accession number WOWW00000000 and MT193824, respectively. MLST, resistance, and virulence associated genes were confirmed using online tools that were provided by the Danish Technical University (DTU) (http://www.genomicepidemiology.org). Annotation was carried out using RAST2 of the PATRIC database (www.particbrc.org). 

### 2.3. Farm Investigation

The farm was re-investigated three months after detecting the isolate 19-AB01443 within the national monitoring. Composite fecal samples (*n* = 28) were taken from the floor of all pens. Additionally, environmental samples (*n* = 17, from i.e., dust, insects, walls, feed) were taken. The samples were analyzed, as previously described, following the EURL method combined with a second enrichment step and additional real-time PCR analysis [12].

## 3. Results

### 3.1. Isolate Characterization

Within the German monitoring on CPE in livestock and food, the *E. coli* isolate 19-AB01443 was detected on chromID^®^ Carba as well as on chromID OXA-48 agar (Biomérieux, Nürtingen, Germany). Broth microdilution showed that the recovered bacteria exhibited a non-wildtype phenotype for some beta-lactams (ampicillin, cefotaxime, cefepime, temocillin), including the tested carbapenems (imipenem, meropenem, ertapenem). However, it was susceptible to all other tested antimicrobials (Supplementary Table S1). Further molecular characterization indicated that the phylogenetic group B1 isolate belonged to ST295 and harbored the *bla*_OXA-48_ gene on a 63,427 bp IncL/M plasmid (pOXA-48EC-1443). Beside *bla*_OXA-48_, no further antimicrobial resistance determinant was located on the plasmid. In addition, the isolate harbored the macrolide resistance gene *mdf(A)* (98.46% nucleotide identity, Acc. no. Y08743), which might be defective due to the sequence alterations, as there was no phenotypic resistance detected (Supplementary Table S1). Hence, only the virulence associated genes *gad* and *ipfA* were detected, which code for a glutamate decarboxylase and a long polar fimbriae component, respectively. Table 1 summarizes the main characteristics of the isolate.

### 3.2. pOXA-48EC-1443 Plasmid

**Figure 1:**
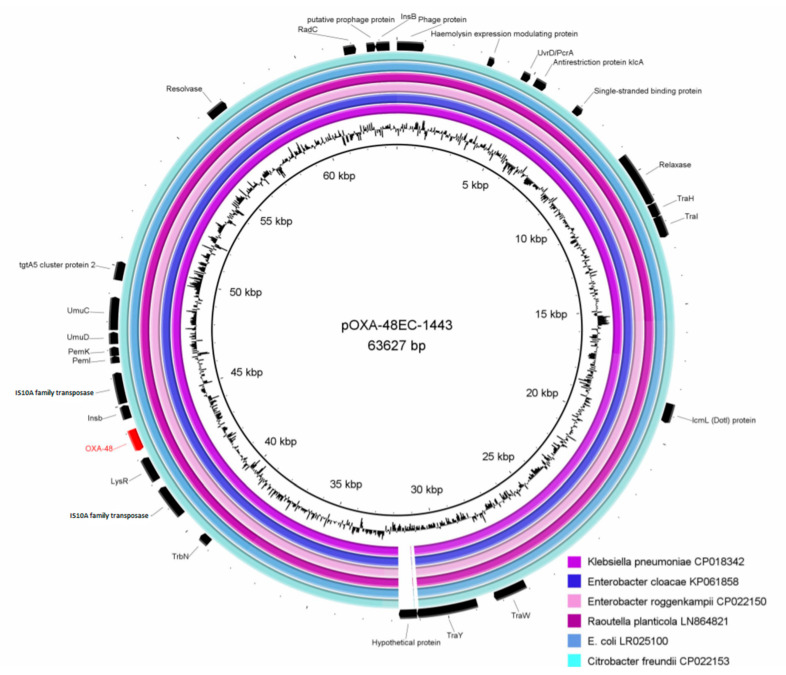
Pairwise alignment of closely related OXA-48-harbouring plasmids from different Enterobacteriaceae to pOXA-48EC-1443. Acc-No. of the plasmid sequences are given in the figure legend. Key features (gene products) of pOXA-48EC-1443 are represented by the black arrows according to their gene orientation. Annotation was carried out using RAST2 of the PATRIC database (www.particbrc.org).

The transmissibility of the pOXA-48EC-1443 plasmid was investigated by filter mating assay. It was highly transmissible by conjugation into a broad range of Enterobacteriaceae (*E. coli, Citrobacter freundii, Enterobacter asburiae*, *Klebsiella oxytoca*, and *Salmonella* Typhimurium). Comparison of the plasmid genome with available pOXA-48-like sequences of GenBank (National Center for Biotechnology Information; NCBI) revealed a high degree of nucleotide identity (>99%) and coverage (94%) among the different plasmid genomes detected in Enterobacteriaceae (e.g., *E. coli*, LR025100; *K. pneumoniae*, CP018342; *Citrobacter freundii*, CP022153; *Raoultella planticola*, LN864821; *Enterobacter roggenkampii*, CP022150 or *Enterobacter cloacae*, KP061858) (Figure 1). The highest concordance (identity: 99.96%, coverage: 94%) was found to the plasmid of *K. pneumoniae* (Acc. no. CP018342.1) that was isolated during a nosocomial outbreak in Lower Saxony (Germany), in 2016. As previously reported, the *bla*_OXA-48_ gene of pOXA-48EC-1443 is associated with the Tn1999 transposon with no other foreign DNA being integrated at this locus [7].

By analyzing the plasmidome of 19-AB01443, another extrachromosomal-replicating element of 4,853 bp was identified. This plasmid exhibited four transfer genes *mobA* to *mobD* and it was mobilized by pOXA-48EC-1443 during conjugation. This plasmid was identical to a plasmid from *Enterobacter hormaechei* strain C15 (CP042491.1) and further highly identical to plasmids from other *Enterobacter* species deposited at GenBank. The respective 4,835 bp plasmid seemed to be acquired independently, as the related strains were not associated with OXA-48 plasmids.

### 3.3. Farm Investigation

Trace-back analyses were conducted to determine whether the isolate might persist in the farm or in the direct environment of the farm to exclude a further introduction into the food chain Therefore, the farm was further investigated in detail three months after the detection of the OXA-48 *E. coli* 19-AB01443 within the CPE monitoring. At that point in time, CPEs could not be detected in fecal (*n* = 28) or environmental samples (*n* = 17) from the farm. This indicated no further occurrence of OXA-48-producing Enterobacteriaceae, which suggests that the isolate was unable to persist or disseminate the plasmid to other enterobacteria on the farm. Up to now, the source of the OXA-48 resistance or the transmission path onto the farm remains unknown. According to the farmer, neither he nor his family or the workers of the farm had taken antimicrobials or had been hospitalized in the last six months prior to the detection of the isolate. 

## 4. Discussion

In Enterobase (http://enterobase.warwick.ac.uk, access: 09.01.2020), a total of 220 *E. coli* isolates of the ST295-type are available. The isolates of this *E. coli* lineage originated from various sources (human, environment, livestock, and wild animals) worldwide and have been detected since 1956. The ubiquitous occurrence of the pOXA-48 (19-AB01443) precluded the identification of the potential reservoir. The isolates of this sequence type were characterized as both pathogenic and non-pathogenic based on their genomic features. Only low pathogenicity of the isolate in our study is assumed based on the few virulence-associated genes detected.

The pOXA-48EC-1443 is closely related to other pOXA-48-like plasmids prevailing in a broad range of Enterobacteriaceae, indicating that the plasmid prototype is able to replicate in a broad range of host bacteria. Isolates of Enterobacteriaceae harboring this plasmid-prototype were recovered from various sources (i.e., human infections or clinical waste water) and from different countries in Europe and the Mediterranean (i.e., Ireland, Portugal, Czech Republic, Switzerland, Germany, France, Turkey, Lebanon, Morocco, Egypt) [7,17]. Despite its broad dissemination, pOXA-48-like plasmids seem to be mostly associated with *K. pneumoniae* [18]. This could be due to the frequent occurrence of *Klebsiella* spp. as causative agents of nosocomial infections that have to be treated with antimicrobials. Accordingly, their relative abundance as isolated OXA-48-producers is higher when compared to commensal Enterobacteriaceae. Although pOXA-48-like plasmids were also identified in isolates from wildlife before, they seem to be predominantly related to humans or human environments [19]. These observations support our hypothesis that introduction of the pOXA-48EC-1443 plasmid into the pig farm might be associated with close contact to a colonized human (farmer, veterinarian, sampling personal) or to a yet unknown environmental source (i.e., rodents, birds, contaminated feed). The true origin of the resistance plasmid remains unclear due to the high transferability of the plasmid between different hosts and the access of the animals to external parts of the stable. 

A permanent colonization of animals at the farm or a repeated introduction into the farm seems unlikely, as no OXA-48-producing Enterobacteriaceae has been detected during the re-investigation of the farm. Pig production on the farm is carried out with a minimum use of antimicrobials due to a special production concept for animal welfare reasons. As pOXA-48EC-1443 provides no further antimicrobial resistance gene, a co-selection of the plasmid using any other antimicrobial substance than beta-lactams would not be likely (Supplementary Table S1). Nevertheless, this example underlines that CPEs can be introduced into the food chain. As confirmed for *bla*_VIM-1_-carrying Enterobacteriaceae, in principle, CPE can persist in animal production if they are adapted to the animals and the farm environment and stabilized by co-expression of further resistance genes [20]. In concordance with the One Health concept, the spread of CPE in humans poses a risk for the animal and food sector, as CPEs may establish there and this might subsequently contribute to a further spread. Currently, our focus is on early CPE detection in livestock including re-testing identified herds to enable future action. This was the first detection of *bla*_OXA-48_ in the German animal production. So far, there is no hint to a livestock reservoir, but the monitoring of carbapenemase producing bacteria in livestock is crucial to notice a potential manifestation early. The transmission of transferable carbapenem resistance through the food chain would pose an additional risk for further spread within the community.

## Figures and Tables

**Table 1:** Summary of the characteristics of the *E. coli* isolate 19-AB01443. Abbreviations: ampicillin—AMP, cefepime—FEP, ertapenem—ETP, cefotaxime—FOT, imipenem—IMP, meropenem—MERO, and temocillin—TEMOCI.

Isolate	Origin	Phenotype	AMR Genes	Phylogenetic Group/MLST	Plasmid Size	Inc- Group
19-AB01443	Pig, fecal sample	AMP, ETP, FEP, FOT, IMP, MERO, TEMOCI	*bla*_OXA-48_	B1 / ST295	63 kb	IncL/M

## Supplementary Materials

The following are available online at https://www.mdpi.com/2076-2607/8/6/855/s1, **Table S1**: MIC values (mg/L) of the isolates 19-AB01443 and transformants.
